# Mechanisms and regulation of organic acid accumulation in plant vacuoles

**DOI:** 10.1038/s41438-021-00702-z

**Published:** 2021-10-25

**Authors:** Xiao-Yu Huang, Chu-Kun Wang, Yu-Wen Zhao, Cui-Hui Sun, Da-Gang Hu

**Affiliations:** National Key Laboratory of Crop Biology; Shandong Collaborative Innovation Center of Fruit & Vegetable Quality and Efficient Production; College of Horticulture Science and Engineering, Shandong Agricultural University, Tai’an, Shandong 271018 China

**Keywords:** Plant molecular biology, Plant signalling

## Abstract

In fleshy fruits, organic acids are the main source of fruit acidity and play an important role in regulating osmotic pressure, pH homeostasis, stress resistance, and fruit quality. The transport of organic acids from the cytosol to the vacuole and their storage are complex processes. A large number of transporters carry organic acids from the cytosol to the vacuole with the assistance of various proton pumps and enzymes. However, much remains to be explored regarding the vacuolar transport mechanism of organic acids as well as the substances involved and their association. In this review, recent advances in the vacuolar transport mechanism of organic acids in plants are summarized from the perspectives of transporters, channels, proton pumps, and upstream regulators to better understand the complex regulatory networks involved in fruit acid formation.

## Introduction

Organic acids influence fleshy fruit acidity and play an important role in regulating osmotic pressure, pH homeostasis, stress resistance, and fruit organoleptic quality^1,2^. While the predominant organic acid in fleshy fruits varies among species, malic and citric acids are the main acids found in most ripe fruits^2–4^. In general, the terms “malate” and “citrate” denote the conjugate base of malic and citric acid, respectively, but are used to represent all physiological forms of these organic acids in plants^5,6^.

**Fig. 1:**
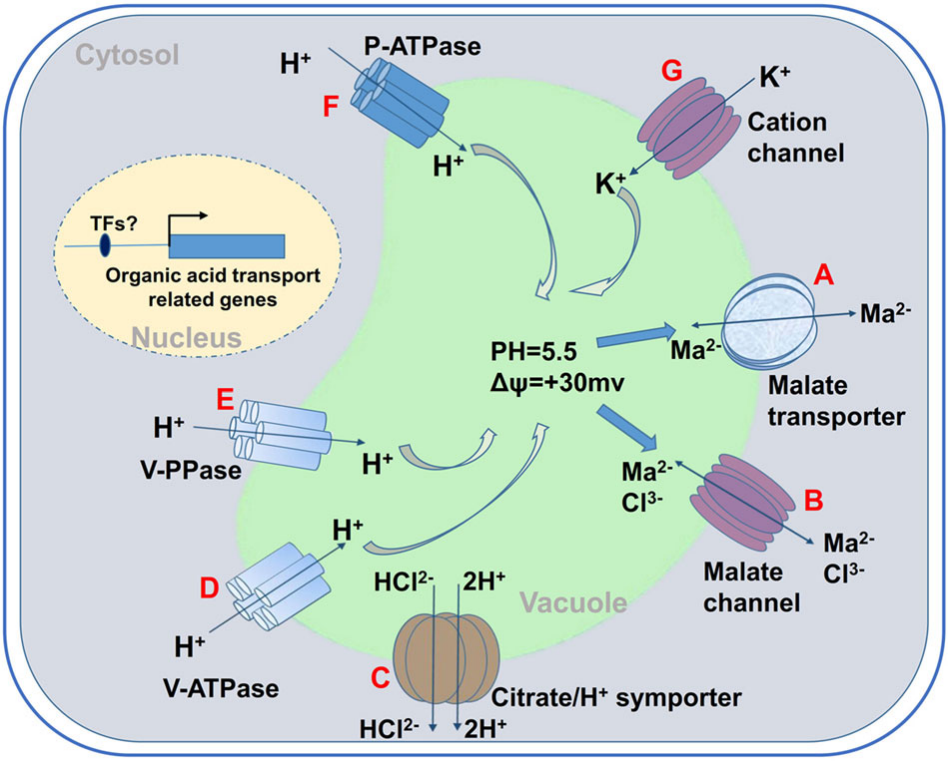
Vacuolar proteins that are involved in the transport of organic acids. The vacuolar transport of organic acids is mainly mediated by channels, carriers, and proton pumps. Carriers catalyze either the transport of a single solute or the coupled transport of two solutes. Carrier proteins can be involved in promoting diffusion and secondary active transport of counter-electrochemical potential gradients by pro-electrochemical potential gradients. Channel proteins are another class of transporters that differ from carrier proteins in that they are simply gated membrane protein channels for diffusion. Channels act as selective holes through which molecules or ions can diffuse across the membrane. Proton pumps catalyze the coupled transport of a solute with a chemical reaction, which allows X- to utilize the energy released by the hydrolysis of ATP or PPi (ΔGATP (or PPi) <0). These mechanisms allow ionic material (X-) to pass through the biofilm and are governed by the general principle of thermodynamics that the change in free energy (ΔG1-2) of the transport reaction must be negative. During the vacuole transport of organic acids: (1) Diffusion includes simple diffusion mediated by channels (**C**) (used to transport some cations such as Na^+^, K^+^, and Ca^2+^) and facilitated diffusion mediated by carriers (**A**, **B**) (which is the main mode of transport of organic acids from the cytoplasm to the vacuole). (2) Primary active transport is mediated by three types of pumps (**D**, **E**, **F**) on the vacuole. Proton entry into the vacuole provides good conditions for the transport of organic acids: acidic vacuolar pH and a positive electric potential gradient. (3) Secondary active transport is mediated by symports (**C**), which are mainly involved in the transport of citrate out of the vacuole

Malate and citrate accumulation in plant cells is mostly attributed to their complicated metabolism and vacuolar storage^2,7^. Several pathways are involved in malate and citrate metabolism in the mesocarp cells of fleshy fruits. Among these, four typical pathways—the tricarboxylic acid cycle in the mitochondrion, the glyoxylate cycle in the glyoxysome, and citrate catabolism and decarboxylation of malate and oxaloacetate in the cytosol—are responsible for malate and citrate metabolism^2^. Although malate and citrate levels in fleshy fruits are altered by their metabolism^7,8^, it appears that their accumulation levels are largely determined by their transport from the cytosol to the vacuole^2,9–11^. Malate and citrate transport across the tonoplast occurs by facilitated diffusion^2^. This process is mediated by multiple vacuolar transporters, ion channels, and carriers, including the tonoplast dicarboxylate transporter (tDT)^12,13^, the channels of aluminum-activated malate transporters (ALMTs) ALMT6 and ALMT9/Ma1^14–18^, and the vacuolar citrate/H^+^ symporter Cit1^19^. Tonoplast proton pumps such as vacuolar-type H^+^-ATPase (V-ATPase), vacuolar-type H^+^-PPase (V-PPase), and P-ATPase (PH1, PH5, etc.) drive the facilitated diffusion of malate and citrate into the vacuole^2,10,20,21^ (Fig. 1). Malate and citrate anions are protonated upon entering an acidic vacuole, and malate and citrate are trapped in the acid form to effectively maintain their concentration gradient across the tonoplast for continuous diffusion into the vacuole^2,10,22^ (Fig. 1).

Malate and citrate accumulation in mesocarp cells is under both genetic and environmental control. Many agronomic studies have investigated the effects of cultural practices, including mineral fertilization^23^, irrigation^24–26^, and thinning^24,25^, and environmental factors such as drought and high temperature^23^ on malate and citrate accumulation. However, how these effects control their accumulation is largely unknown. In this review, recent advances in the vacuolar transport mechanism of organic acids in plants have been outlined from the aspects of transporters, channels, proton pumps, and upstream regulators to enhance the understanding of the complex regulatory networks of fruit acid formation.

## Vacuolar transporters and channels play a central role in organic acid transport

### Vacuolar transporters and channels play a central role in malate transport

Currents of organic acids are strongly inward rectified, thus promoting the transfer of organic acids from the cytosol to the vacuole^27,28^. These currents through the membranes also have the characteristics of anion selectivity and activation under high membrane potential. Remarkably, vacuolar transporters and channels play a central role in organic acid transport. Malate transport to vacuoles is catalyzed by at least one transporter and several channels^29^. Malate channel activity is reduced after *AttDT* is knocked out in *Arabidopsis*, indicating that AttDT has malate transport activity and is an essential malate channel^13^.

The ALMT gene family plays a major role in malate transport. ALMT family genes encoding transmembrane proteins as anion channels perform various functions, including inorganic anion transport, aluminum resistance, mineral nutrition absorption, microbial interaction, fruit acid formation, light response, and seed development^30^. TaALMT1, the first identified malate transporter, is activated by the trivalent aluminum cation in acidic soil and releases the malate anion into the apoplast in wheat plants. Thus, apoplastic malate chelates aluminum cations to reduce damage to cell walls, membranes, and other cellular components^31,32^. Other ALMTs perform similar functions in *Arabidopsis*, rapeseed, rye, soybean, and alfalfa^33,34^. Some of them can also transport organic acids through different membranes. For example, AtALMT1 is a malate-permeable channel expressed in *Arabidopsis* roots that plays a vital role in resistance to aluminum by secreting malate^35^. AtALMT3 is a malate transporter involved in low phosphorus-induced malate secretion in *Arabidopsis* and is mainly located in the plasma membrane^36^. In rice, *OsALMT4* encodes a malic acid-permeable anion channel in the plasma membrane^37^. Both *SlALMT4* and *SlALMT5* are expressed in the endoplasmic reticulum of tomato, and *SlALMT5* is also expressed in the intima of tomato; both can transport malate under normal electrophysiological conditions in the cell^38^. In mature tomato seeds, *SlALMT5* overexpression showed high concentrations of malate and citrate^37^. AtALMT6 present in the tonoplast is a calcium-activated malate transporter that mediates malate transport from the cytosol to the vacuole in guard cells. Its activity is regulated by vacuolar pH and the cytoplasmic malate concentration^17^.

Among the ALMT family members, *ALMT9* is the most widely studied gene. *AtALMT9* is a member of the ALMT subfamily in *Arabidopsis*. It is widely expressed in plant cells, such as mesophyll and guard cells, and is insensitive to cytoplasmic Ca^2+^, but it can be activated by cytoplasmic malate. After *AtALMT9* is knocked out, the malate current in the vacuole is inhibited, indicating that AtALMT9 mainly functions as a malate channel^14,15,39^. VvALMT9, an AtALMT9 homolog in grape, is a vacuolar malate channel that mediates malate and tartrate accumulation in the vacuoles of grape cells. It also has a stronger ability to transport tartrate than AtALMT9^40^. *SlALMT9* is a major quantitative trait locus on chromosome 6 in tomato that is responsible for fruit malate accumulation and is considered a malate candidate gene for genotypic variation of fruit quality^30^. In apple, two *ALMT9* homologous genes, *Ma1* and *Ma2*, in the Ma region of the genome are the major sites contributing to malate accumulation^41,42^.

ALMT9 is different from other ALMT family members, and much attention has been focused on malate transport mainly because it is the major contributor to malate accumulation; the unique C-terminal domain structure of ALMT9 determines its function^15,43–45^. In *Arabidopsis*, ALMT9 is a tetramer, and the TMa5 domain of each subunit contributes to the formation of anion channel pores. TMa1 and TMa2 are connected by salt bridges, and these special structures are related to the function of ALMT9^39^. A 1455-bp mutation in the *Ma1* open reading frame resulted in an early termination codon that truncated 84 amino acids from the C-terminus of the protein, which may be the direct cause of low acid formation in apple^41^. This phenomenon suggests that the C-terminal structure of ALMT9 may be closely related to its function in *Arabidopsis*. In comparison, apple has two ALMT homologous genes—*Ma1* and *Ma2*. Interestingly, only the expression of *Ma1*, a potential gene for the fruit acidity trait, positively correlated with the fruit acidity level. The locus consists of two alleles—*Ma1* and *ma1*. mal is a truncated protein directly associated with the low malate phenotype^46^. Both Ma1 and ma1 are located on the tonoplast. In plant cells, Ma1 has higher malate transport activity than ma1 because of its highly conserved C-terminal domain structure. Therefore, the highly conserved C-terminal domain structure in ALMTs is essential for the normal function of Ma1, and any truncation, either natural or artificial, significantly reduces the malate transport activity of this conserved domain^16^.

In addition to affecting the function of ALMT9 through changes in its C-terminal domain structure, there are other ways to determine the malate transport function of ALMT9 in plants. For example, in tomato, a 3-bp indel in the *SlALMT9* promoter region led to a high malate phenotype^18^.

### Vacuolar transporters and channels play a crucial role in citrate transport

Unlike that for malate, citrate accumulation is less affected by vacuolar storage control and is much less constrained by thermodynamic conditions. In most fleshy fruits, vacuolar uptake of citrate trianions possibly occurs by facilitated diffusion through malate channels^2^. It appears that the transport of citrate to the vacuole is much easier than that of malate at their optimal cytosolic concentration. This is because the thermodynamic conditions are more favorable for citrate uptake than for malate uptake at any vacuolar pH and electric potential gradient. However, the rate at which citrate passes through the malate channel under the control of cytoplasmic concentration suggests that citrate accumulation in vacuoles is mainly controlled by metabolism^2,47^. Citrate transport from the cytosol to the vacuole is accompanied by a large influx of protons^48,49^. This proton influx leads to vacuolar acidification and provides a strong driving force for increased vacuolar uptake of citrate, thereby maintaining the vacuolar buffering capacity and acidic pH environment^2,19,50^. This process is mainly mediated by V-ATPase^48,49^. Citrate transport to the vacuole is competitively inhibited by other organic acids, such as malate, because organic acids cross the tonoplast by the same channels or transporters^6^. Citrate accumulation and vacuolar acidification are tightly regulated throughout fruit development and vary among citrate-rich fruit varieties. Usually, lower vacuolar pH values result in higher citrate accumulation in vacuoles^2,51^.

Mechanisms for citrate transport from the cytosol to the vacuole have been elucidated by the manipulation of anion channels and ATP-dependent transporters^5,6,19,52^. AttDT may transport citrate to the vacuole; however, it is not the main tonoplast citrate carrier in *Arabidopsis*^13^. In citrus plants, the citrate transporter 1 gene *CsCit1*, encoding a novel vacuolar citrate/H^+^ symporter, mediates CitH^2−^- and CitH^2−^-dependent H^+^ efflux from the vacuole and maintains vacuolar acidic pH and citric acid homeostasis^19^. The multidrug and toxic compound extrusion (MATE) gene *AtMATE*, encoding an Al-activated citrate transporter in *Arabidopsis*, contributes to aluminum-activated root citrate exudation^53^. In addition, Lin et al.^54^ showed that the dicarboxylate carrier CitDIC and the cation/H^+^ exchanger CitCHX are involved not only in citrate degradation during fruit development but also in hot air-triggered citrate reduction after harvest. Nevertheless, studies on the regulation of citrate transport are largely limited in comparison with those on the regulation of malate transport.

## Proton pumps provide favorable conditions for organic acid transport to vacuoles

Apart from the large number of transporters and channels in the tonoplast, proton pumps also help in the transport of organic acids from the cytosol to the vacuole. Proton pumping into the vacuole often produces an acidic vacuolar pH and positive electric potential gradient^2^. All three proton pumps, V-ATPase, V-PPase, and P-type ATPase, are present in the vacuoles of fruit cells.

### Widely characterized V-ATPase and V-PPase in several plant species

Vacuoles contain two proton pumps—V-ATPase and V-PPase. Both proton pumps can effectively acidify the vacuolar lumen. V-ATPase and V-PPase are localized to the tonoplast, but their contributions to proton pumping vary during fruit development^22,55–58^. V-ATPase is the primary proton pump in vacuoles, but V-PPase has higher tonoplast activity than V-ATPase in some crassulacean acid metabolism species^59^. V-PPase is enriched during the early fruit developmental stage, but its function decreases and V-ATPase dominates during the fruit ripening stage^2,60^. Maeshima^61^ proposed that the high V-PPase activity in young tissues is an inhibitor of several polymerization reactions, such as RNA and starch syntheses, by scavenging more pyrophosphate (PPi). In mature fruits, PPi production may decrease since its synthesis gradually decreases, whereas ATP is constantly supplied by cellular respiration.

The main functions of V-ATPase are to transport protons to vacuoles by ATP hydrolysis and to generate pH and potential energy gradients inside and outside vacuoles to provide suitable conditions for the transport of metabolites such as organic acids. V-ATPase is a highly conserved and sophisticated complex that contains the peripheral subcomplex V_1_ and the membrane-bound subcomplex V_0_^62,63^. Subcomplex V_1_ is located in the cytosol and comprises eight subunits from A to H, which are mainly responsible for ATP hydrolysis. In contrast, subcomplex V_0_ is embedded in the tonoplast and comprises six subunits—a, b, c′ (not present in plants), c′′, d, and e—which are mainly responsible for H^+^ translocation from the cytosol to the vacuolar lumen^64^. The functions of V-ATPase are quite diverse and have been well described. V-ATPase is involved in energizing secondary transport, vacuolar acidification, ion homeostasis, and stress tolerance in several plant species^62,63,65–67^.

Compared to V-ATPase, V-PPase is a homodimer of a single polypeptide that uses the energy of the PPi phosphoanhydride bond to drive proton transport across the tonoplast; unlike other proton pumps, PPi is its only source of energy^68,69^. Similar to V-ATPase, V-PPase performs diverse functions, such as secondary metabolite transport and stress tolerance^70–73^.

### P-type ATPases are involved in proton transport and vacuolar acidification

P-type ATPases comprise a novel vacuolar proton pump family involved in proton transport and vacuolar acidification, distinct from V-ATPase and V-PPase. These ATPases are primary transporters that are energized by ATP hydrolysis with a series of specificities for small cations and phospholipids and are characterized structurally as having a single catalytic subunit, 8–12 transmembrane segments, N and C termini exposed to the cytoplasm, and a large central cytoplasmic domain, including the phosphorylation and ATP binding sites^74,75^. In plants, these ATPases are composed of five major evolutionarily associated subfamilies, P1–P5, which are classified by the ions they transport^75^. Among them, P3 subfamily ATPases are responsible for energizing the electrochemical gradient used as the driving force of secondary transporters^74,76^.

In petunia, *PH5* encodes a tonoplast-localized P_3A_-ATPase proton pump that interacts with the P_3B_-ATPase PH1 complex to acidify the vacuolar lumen of petal cells, thereby affecting petal color^42,76,77^. Interestingly, PH5 is the only P_3A_ subfamily ATPase that is located on the tonoplast (all others are located on the plasma membrane) and independently exhibits strong proton transport activity. In contrast, PH1 does not have proton transport activity but is necessary for maintaining proton pump activity. It can form a heteromeric pump with PH5 that hyperacidifies the central vacuole of epidermal cells in petunia petals^42,77^. The interaction between PH1 and PH5 can reduce the stoichiometric value of H^+^/ATP from 1.0 to 0.5 H^+^/ATP, resulting in vacuolar hyperacidification^78^. In addition, protein trafficking from vacuolinos to the central vacuole is impaired by the misexpression of either the *PH1* or the *PH5* component of the heteromeric PH1–PH5 pump^79^.

The regulation of pH by P_3A_-ATPase/P_3B_-ATPase exists not only in petunia but also in other angiosperms. However, independent losses of these homologs occur in many angiosperms^76^. PH5 homologs are found in both angiosperms and gymnosperms. These homologs have also been found in some fruits, and their mechanism of action is currently being explored. In apple, *MdPH1* and *MdPH5* have been identified and shown to be involved in vacuolar acidification and malate accumulation^9^. Their homologs, *CitPH1* and *CitPH5*, are expressed in the fruits of sour lemon, orange, pummelo, and rangpur lime; however, their expression is significantly decreased in low acid varieties^80^. Additionally, a candidate gene for fruit acidity in apple, *Ma10*, encodes a P_3A_-ATPase proton pump, which promotes malate uptake into the vacuole and facilitates vacuolar acidification^21^.

To function, P-type ATPases either require other protein complexes or influence the activities of these protein complexes. Changes in PH1–PH5 activity caused by lower vacuolar pH triggered the collapse of the V-ATPase complex or caused other structural changes^81^. However, the regulatory mechanism between P-type ATPase and V-ATPase activity remains elusive. In addition, only P-type ATPase, but not V-ATPase, is used to acidify vacuoles in some specific tissues, such as petunia petals. In addition, PH1 activates PH5 through a still unknown mechanism.

## Reverse transporters promote electrochemical gradient formation inside and outside vacuoles

In addition to malate transporters, channels, and proton pumps, some cation channels or cation/H^+^ reverse transporters (Fig. 1) also contribute to the formation of an electrochemical gradient inside and outside the vacuole. The transport of Na^+^ or K^+^ is an example of secondary transporter assistance. Na^+^/H^+^ (NHX) and K^+^/H^+^ exchangers are responsible for vacuolar alkalization, thereby changing the petal color from purple to blue^82^. *NHX* gene expression is associated with a change in vacuolar pH. For example, the Na^+^/H^+^ antiporters NHX1 and NHX2 control K^+^ homeostasis and vacuolar pH in *Arabidopsis*^83^. Plant NHX proteins passively exchange H^+^ along with K^+^ and Na^+^, consuming the H^+^ gradient during the process^84^. The protein InNHX1 mediates H^+^ outflow from the vacuolar lumen, and the other NHX proteins, including InNHX2, also participate in vacuolar alkalinization and changing petal color by using the K^+^ gradient rather than the H^+^ gradient in Japanese morning glory^85,86^. In addition, the increased expression of the MATE family gene *HvAACT1* in wheat and barley was associated with increased citrate efflux from root apices^87^.

## Upstream regulators that are involved in vacuolar transport of organic acids

### Transcriptional regulation of vacuolar transport of organic acids

Regulation of organic acid transporters and proton pumps involves a complex gene regulatory network. Transcriptional regulation is one of the most common and direct ways to regulate malate follicle transporters and proton pumps. These include transcription factors such as MYB, bHLH, WRKY, and ERF family members (Table 1). These transcription factors play an important role in organic acid transport. Specifically, transcription factors can activate or inhibit the expression of transporters and proton pumps, eventually promoting or impeding the whole process of organic acid transport.

**Table 1:** Upstream regulators that are involved in vacuolar transport of organic acids

Family classification	Protein name	Positive/negative regulator	Regulatory modes	Plant species
MYB transcription factors	MdMYB1	Positive	Transcriptional regulation	*Malus domestica* Borkh
	MdMYB73	Positive		*Malus domestica* Borkh
	CrMYB73	Positive		*Citrus reticulata* Blanco
	VvMYB5a	Positive		*Vitis vinifera*
	VvMYB5b	Positive		*Vitis vinifera*
	PhPH4	Positive		*Petunia hybrid*
	GmPH4	Positive		*Glycine Max*
WRKY transcription factors	PhPH3	Positive		*Petunia hybrid*
	VvWRKY26	Positive		*Vitis vinifera*
	SlWRKY42	Negative		*Solanum lycopersicum*
	AtWRKY46	Negative		*Arabidopsis thaliana*
bHLH transcription factors	CitAN1	Positive		*Citrus reticulata* Blanco
	VvMYC1	Positive		*Vitis vinifera*
	MdCIbHLH1	Positive		*Malus domestica* Borkh
	MdbHLH3	Positive		*Malus domestica* Borkh
	MdbHLH49	Negative		*Malus domestica* Borkh
ERF transcription factor	CitERF13	Positive		*Citrus reticulata* Blanco
WD40 protein	CitAN11	Positive		*Citrus reticulata* Blanco
Protein kinases	AtWNK	Positive	Post-translational modification	*Arabidopsis thaliana*
	MdSOS2L1	Positive		*Malus domestica* Borkh
	AtPKA	Positive		*Arabidopsis thaliana*
	MdHXK1	Positive		*Malus domestica* Borkh
E3 ligase	MdSIZ1	Positive		*Malus domestica* Borkh
	MdPUB29	Negative		*Malus domestica* Borkh
	MdCOP1	Negative		*Malus domestica* Borkh
Small auxin-up RNA	MdSAUR37	Positive		*Malus domestica* Borkh
Protein phosphatase	MdPP2CH	Negative		*Malus domestica* Borkh
BTB-BACK-TAZ domain protein	MdBT2	Negative		*Malus domestica* Borkh

**Fig. 2:**
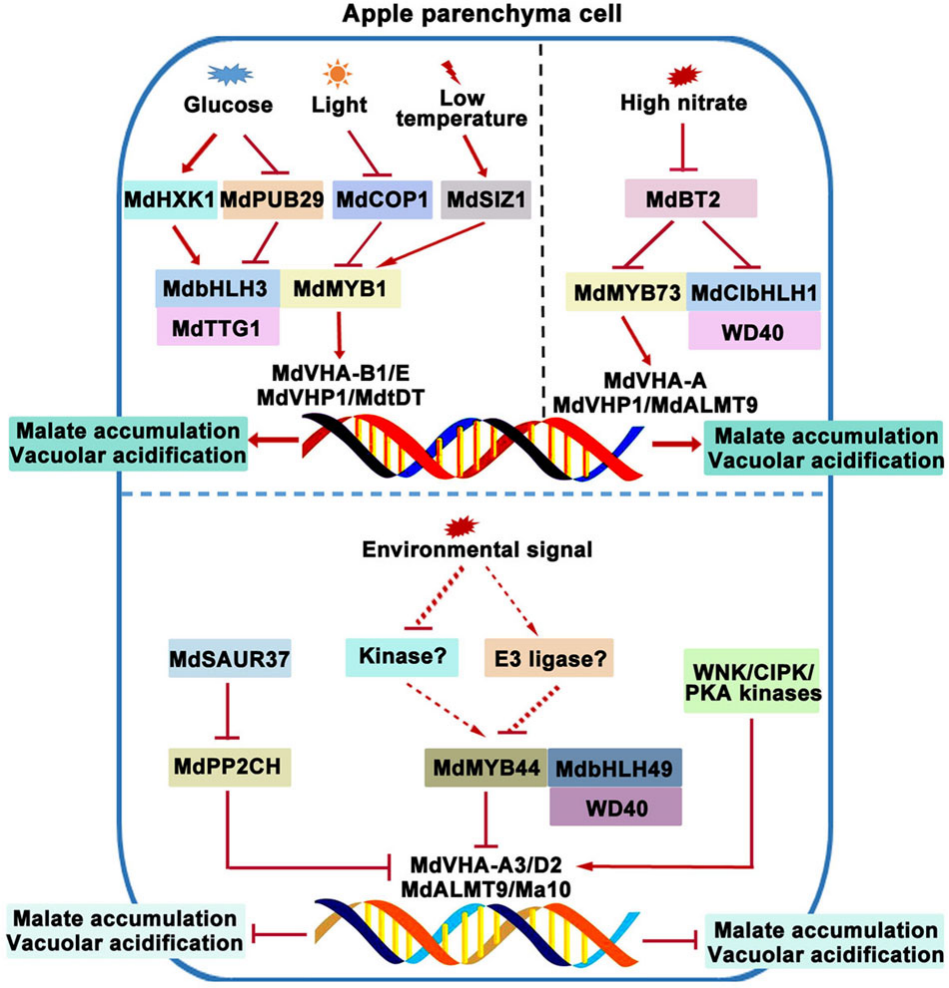
Upstream regulators that are involved in the vacuolar transport of malate and vacuolar acidification in apple parenchyma cells. The graphs above the blue dotted line show the positive regulators of MYB-bHLH-WD40 (MBW) complexes, including MdMYB1-MdbHLH3-MdTTG1 and MdMYB73-MdCIbHLH1-WD40, that are involved in malate accumulation and vacuolar acidification; the stabilities of these two MBW complexes can be affected by posttranslational modifications, such as phosphorylation and ubiquitination, in response to environmental stimuli. The graphs below the blue dotted line reveal the negative regulators of the MBW complex or protein phosphatases and kinases involved in malate accumulation and vacuolar acidification

The MYB transcription factor family is one of the largest transcription factor families in plants. Most members of this family, which is involved in organic acid transport, are plant-specific R2R3-MYB transcription factors, which also play important roles in plant growth and development and in biotic and abiotic responses^88^. In terms of fruit quality, MYB transcription factors can directly regulate the transcriptional activities of organic acid transporters and metabolic enzymes, resulting in organic acid accumulation and vacuolar acidification in fleshy fruit cells. In apple, MdMYB1, MdMYB44, and MdMYB73 are distant relatives^89,90^. MdMYB1, MdMYB44, and MdMYB73 can regulate the transcriptional activities of the malate transporter and proton pump to control malate accumulation and vacuolar acidification^9,91,92^ (Fig. 2). However, MdMYB1 and MdMYB73 are positive regulators, whereas MdMYB44 is a negative regulator. Moreover, their downstream target genes are different. The direct downstream target genes of MdMYB1 are the V-ATPase subunit genes *MdVHA-B1* and *MdVHA-E*, V-PPase gene *MdVHP1*, and tDT gene *MdtDT*^91^ (Fig. 2). In contrast, MdMYB73 directly activates the expression of the V-ATPase subunit gene *MdVHA-A*, V-PPase gene *MdVHP1*, and ALMT gene *MdALMT9* but not the expression of the genes *MdVHA-B1*, *MdVHA-E*, and *MdtDT*^9^ (Fig. 2). However, MdMYB44 negatively regulates malate accumulation in apple by repressing the promoter activity of the V-ATPase subunit genes *MdVHA-A3* and *MdVHA-D2*, P-type ATPase gene *Ma10*, and ALMT gene *MdALMT9*^92^ (Fig. 2). In citrus plants, *CrMYB73*, homologous to apple *MdMYB73*, confers an increase in citrate accumulation, but its downstream target genes are unknown^93^. In addition, petunia PhPH4 is an R2R3-MYB transcription factor that plays a similar role as grape VvMYB5a and VvMYB5b in the regulation of citrate accumulation; both can activate the expression of the downstream genes *PH1* and *PH5*, thus acidifying vacuoles^94–96^. Similarly, the R2R3-MYB transcription factor GmPH4 is also involved in vacuolar acidification by directly regulating the expression of the P_3A_-type ATPase gene *GmPH5* in soybean petals^97^.

Plant-specific transcription factors containing WRKY domains are also important for organic acid transport and vacuolar acidification^98,99^. WRKY transcription factors can regulate downstream transporters by binding to specific W-box *cis*-elements in promoters. Fittingly, W-box *cis*-elements are highly enriched in the promoters of ALMT family genes, which are major contributors to malate transport and vacuolar acidification. For example, WRKY46 functions as a transcriptional repressor of *ALMT1* by directly binding the specific W-box to its promoter, thus regulating aluminum-induced malate secretion in *Arabidopsis*^100^. An indel in the *SlALMT9* promoter disrupts a W-box binding site, which prevents the binding of the WRKY transcription repressor SlWRKY42, thereby decreasing the repression of *SlALMT9* expression and facilitating high malate accumulation in tomato^18^.

**Fig. 3:**
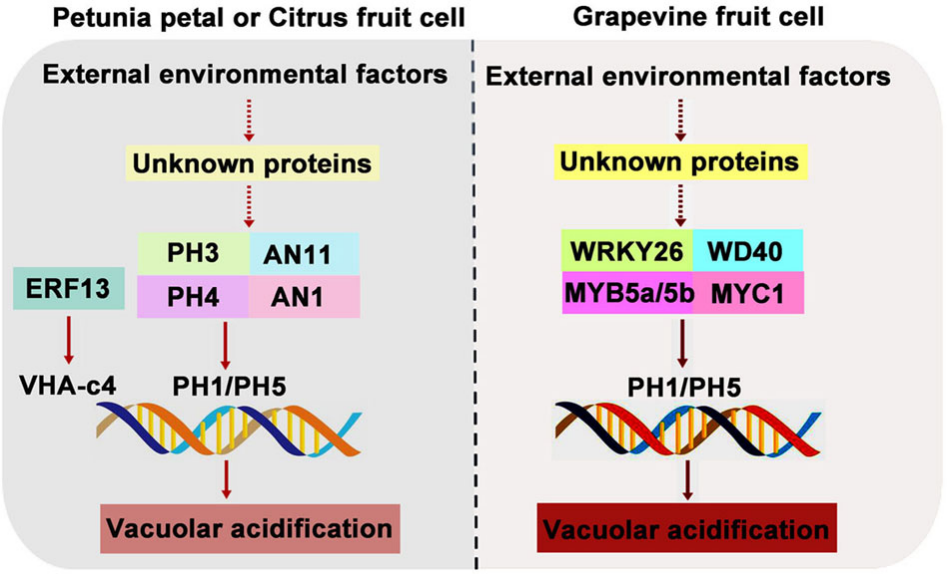
Upstream regulators that are involved in vacuolar acidification in petunia petal, citrus and grapevine fruit cells. The graph to the left of the black dotted line shows the WRKY- MYB-bHLH-WD40 (WMBW) complex and ERF transcription factor that are involved in vacuolar acidification in petunia petal and citrus fruit cells. The graph to the right of the black dotted line reveals the WMBW complex that is involved in vacuolar acidification in grapevine fruit cells

Apart from MYB and WRKY transcription factors, other transcription factors, such as bHLH and ERF, are also involved in the regulation of organic acid accumulation and vacuolar acidification. By analyzing the fruits of sour lemon, orange, pummelo, and rangpur lime, Strazzer et al.^80^ found that inactivating mutations in *CitAN1*, which encodes a bHLH transcription factor, led to the decreased expression of *CitPH1* and *CitPH5*, thereby resulting in the vacuolar hyperacidification of juice vesicle cells (Fig. 3). In apple, the bHLH transcription factor MdbHLH3 directly regulates the expression of the cytosolic malate dehydrogenase gene *MdcyMDH* to coordinate carbohydrate allocation and malate accumulation^101^. The citrus transcription factor CitERF13 regulates citrate accumulation by directly activating the vacuolar proton pump gene *CitVHA-c4*^102^ (Fig. 3), whereas the CitWRKY1-CitNAC62 complex contributes to citric acid degradation in citrus fruits, potentially via the modulation of *CitAco3*^103^. An EIN3-like transcription factor is considered the regulator of ALMT1-like proteins in apple^104^.

Additionally, these transcription factors can form a complex and cooperate in acidifying vacuoles, a function they cannot perform independently. The MYB-bHLH-WD40 (MBW) complex performs an important function in organic acid accumulation and vacuolar acidification. As mentioned earlier, the MYB transcription factors MdMYB1, MdMYB44, and MdMYB73 affect malate accumulation and vacuolar acidification by regulating the activities of vacuolar proton pumps and malate transporters in apple^9,91,92^ (Fig. 2). These regulatory activities depend on MBW complex formation. MdbHLH3, MdbHLH49, and MdCIbHLH1 interact with MdMYB1, MdMYB44, and MdMYB73, respectively, and enhance corresponding MYB transcription factor activities, further regulating the activities of downstream genes, including malate transporters and vacuolar proton pumps^9,91,92,105^ (Fig. 2). In petunia petals, the PH4 (MYB)-AN1 (bHLH)-AN11 (WD40) complex controls vacuolar acidification by directly regulating *PH5* transcription^106^ (Fig. 3). Similarly, *CitPH1* and *CitPH5* are transcriptionally activated by the CitPH4 (MYB)-CitAN1 (bHLH)-CitAN11 (WD40) complex and cause hyperacidification of citrus fruits^80^ (Fig. 3). Interestingly, the WRKY transcription factor forms a more complicated WMBW complex with the MBW complex to affect vacuolar acidification. For example, *PH3* is a target gene of the PH4-AN1-AN11 complex; it encodes a WRKY protein that can bind to AN11 and is required for the transcriptional activation of *PH5* in petunia petals as part of a feed-forward loop with the PH4-AN1-AN11 complex^78,106–108^ (Fig. 3). Likewise, the CitPH3 (WRKY)-CitPH4 (MYB)-CitAN1 (bHLH)-CitAN11 (WD40) complex in citrus plants regulates the expression of its target genes *CitPH1* and *CitPH5* to control fruit acidity and titratable acid content^80^ (Fig. 3). In grape, VvWRKY26 enhances the expression of the target genes *VvPH1* and *VvPH5* induced by the VvMYB5a/b-VvMYC1 (bHLH)-WD40 (unknown) complex^94,109^ (Fig. 3). Collectively, these reports suggest that the transcriptional regulation of the vacuolar transport of organic acids is a complex regulatory process in plants, especially in fleshy fruits.

### Posttranslational modification mediates the vacuolar transport of organic acids

Posttranslational modification, such as phosphorylation or dephosphorylation and ubiquitination, plays an essential role in the vacuolar transport of organic acids. Remarkably, posttranslationally modified proteins can be either proton pumps and organic acid transporters or their upstream transcriptional regulators.

Protein kinases, such as WNK, CIPK, and PKA, modulate organic acid accumulation and vacuolar acidification by directly phosphorylating different V-ATPase subunits^110–113^ (Fig. 2). MdPP2CH of the protein phosphatase 2CH subfamily inactivated three vacuolar H^+^-ATPases (i.e., MdVHA-A3, MdVHA-B2, and MdVHA-D2) and one ALMT (i.e., MdALMTII) via dephosphorylation and reduced fruit malate accumulation. Its dephosphatase activity was inhibited by the small auxin-up RNA MdSAUR37 in apple^114^ (Fig. 2). In addition, Ucc1 (ubiquitination of citrate synthase in the glyoxylate cycle) is a recently characterized F-box protein that promotes the proteasomal degradation of citrate synthase 2 (Cit2) in the glyoxylate cycle^115^. Other enzymes associated with the glyoxylate cycle or the gluconeogenic pathways, such as malate dehydrogenase (Mdh2), isocitrate lyase (Icl1), and phosphoenolpyruvate carboxykinase (Pck1), are also regulated by either the vacuole import and degradation pathway or the glucose-induced degradation-deficient pathway^115^. These studies provide important insights into the transcriptional regulation and direct posttranslational modification of organic acid-related transporters, proton pumps, or key enzymes.

In addition to direct posttranslational modification of proton pumps and organic acid transporters in fleshy fruits, posttranslational modification of their upstream transcriptional regulators is also common. Among these modifications, the posttranslational modification of the MBW complex is the most studied in fleshy fruits, especially in apple (Fig. 2). Apple MdCOP1 ubiquitin E3 ligase interacts with MdMYB1 to regulate malate accumulation and vacuolar acidification^91,116^. The glucose sensor MdHXK1 and high glucose-inhibited U-box-type E3 ubiquitin ligase MdPUB29 phosphorylate and ubiquitinate MdbHLH3, respectively, affecting vacuolar acidification and malate concentration by regulating their downstream malate-associated genes in apple^91,105,117,118^. The BTB-BACK-TAZ domain protein MdBT2-mediated ubiquitination of MdCIbHLH1 and MdMYB73 negatively regulates malate accumulation and vacuolar acidification^119,120^. The small ubiquitin-like modifier E3 ligase MdSIZ1 targets MdbHLH104 to regulate plasma membrane H^+^-ATPase activity and proton efflux^121^ and acidifies vacuoles by sumoylating MdMYB1 and its downstream malate-related genes^91,122^.

## Conclusions and perspectives

Organic acids affect fruit quality and participate in the evolution and reproduction of fleshy fruits^123^. The transport of organic acids from the cytosol to the vacuole and their subsequent storage and potential reutilization are complex processes. Although the vacuolar transport mechanism of organic acids has been partially elucidated in some species, more studies are needed to explore and identify the transporters, proton pumps, and upstream regulators of organic acids that are responsible for organic acid accumulation and vacuolar acidification. Isolation of vacuole and tonoplast proteins, liquid chromatography–tandem mass spectrometry assays, and functional identification are effective methods to explore the mechanisms associated with organic acid accumulation and vacuolar acidification. In conclusion, further studies on the identification of organic acid-associated proteins, the regulation of their function, and the additional roles of organic acids in fruit physiology are needed in the future.
